# Conserved Transcriptome Features Define Prepubertal Primate Spermatogonial Stem Cells as A_dark_ Spermatogonia and Identify Unique Regulators

**DOI:** 10.3390/ijms24054755

**Published:** 2023-03-01

**Authors:** Anukriti Singh, Brian P. Hermann

**Affiliations:** Department of Neuroscience, Developmental and Regenerative Biology, The University of Texas at San Antonio, 1 UTSA Circle, San Antonio, TX 78249, USA

**Keywords:** spermatogonia, stem cells, prepubertal testis, non-human primate, human, A_dark_, A_pale_

## Abstract

Antineoplastic treatments for cancer and other non-malignant disorders can result in long-term or permanent male infertility by ablating spermatogonial stem cells (SSCs). SSC transplantation using testicular tissue harvested before a sterilizing treatment is a promising approach for restoring male fertility in these cases, but a lack of exclusive biomarkers to unequivocally identify prepubertal SSCs limits their therapeutic potential. To address this, we performed single-cell RNA-seq on testis cells from immature baboons and macaques and compared these cells with published data from prepubertal human testis cells and functionally-defined mouse SSCs. While we found discrete groups of human spermatogonia, baboon and rhesus spermatogonia appeared less heterogenous. A cross-species analysis revealed cell types analogous to human SSCs in baboon and rhesus germ cells, but a comparison with mouse SSCs revealed significant differences with primate SSCs. Primate-specific SSC genes were enriched for components and regulators of the actin cytoskeleton and participate in cell-adhesion, which may explain why the culture conditions for rodent SSCs are not appropriate for primate SSCs. Furthermore, correlating the molecular definitions of human SSC, progenitor and differentiating spermatogonia with the histological definitions of A_dark_/A_pale_ spermatogonia indicates that both SSCs and progenitor spermatogonia are A_dark_, while A_pale_ spermatogonia appear biased towards differentiation. These results resolve the molecular identity of prepubertal human SSCs, define novel pathways that could be leveraged for advancing their selection and propagation in vitro, and confirm that the human SSC pool resides entirely within A_dark_ spermatogonia.

## 1. Introduction

Cancer survivors are often faced with negative side-effects of their life-saving treatments, which impact their quality of life and one of the most devastating is the loss of spermatogenesis and the resulting permanent infertility [1]. Spermatogenesis is the process by which the progeny of diploid spermatogonial stem cells (SSCs) terminally differentiate to produce mature spermatozoa in the testis. For adult patients, this infertility risk of chemotherapy and radiotherapy can be mitigated by cryopreserving sperm recovered from an ejaculate for future use in medically-assisted reproductive technologies, like IVF and ICSI [2,3,4]. Prepubertal patients who are not yet producing sperm, however, cannot take advantage of this standard of care [5]. One experimental fertility preservation option that may address the challenges of prepubertal boys is cryopreservation of testicular tissue that contains SSCs prior to commencing gonadotoxic treatments [6,7,8]. The proof of principle that cryopreserved testicular tissue can be used to generate sperm using SSC transplantation has been established in a variety of species, including non-human primates [9,10]. While harvesting testicular tissue for future SSC transplantation into the testis of infertile recipients is a promising approach, utilizing this technology in humans is limited by the lack of exclusive biomarkers that can unequivocally identify therapeutic SSCs, and the lack of knowledge about their development. Moreover, testicular biopsies from prepubertal patients are of limited size and may not contain sufficient SSCs to produce robust spermatogenesis after transplantation. It has been estimated that a 1300-fold increase in SSCs is required to achieve sufficient colonization in a clinical human application [11], and thus, it may be necessary to expand SSCs in culture. Initially, attempts to culture human SSCs were derived from advances in rodent models [12,13]. Subsequently, studies have tested feeder-based conditions utilizing human embryonic stem cells-derived fibroblasts, human Sertoli cells, or an extracellular matrix such as laminin, gelatin, and hydrogel [14,15,16,17,18]. However, the lack of the ability to functionally identify SSCs and the lack of exclusive biomarkers have made it difficult to assess and repeat these experiments. Therefore, future attempts to propagate human SSCs in vitro would benefit from a better understanding of their fundamental characteristics and in vivo kinetics. 

Functionally, SSCs balance self-renewal with the initiation of differentiation to maintain fertility throughout the male reproductive lifespan [19]. Clermont et al. proposed one of the earliest models of SSC self-renewal called the A_0_/A_1_ model based on ^3^H-thymidine-labeled whole mount preparations of rat seminiferous tubules [20,21,22]. According to this model, rodent testes contain two types of stem cells, reserve (A_0_) and active (A_1_). A_0_ reserve stem cells exist as largely quiescent singles and pairs of type-A spermatogonia that do not contribute to ongoing spermatogenesis and only become active upon the loss of spermatogenesis due to gonadotoxic insult [23]. The active stem cell pool (A_1_) undergoes clonal expansion, giving rise to the differentiating A_1_ to A_4_ spermatogonia and stem cell renewal is accomplished by fragmentation of A_2_, A_3_, and A_4_ spermatogonial clones [23]. Although the A_0_/A_1_ model was largely supplanted by three new models describing the identity and kinetics of rodent SSCs [22,24,25,26,27,28], concepts reminiscent of Clermont’s ideas are still incorporated in our current understanding. 

The prevailing “A_single_ model” categorizes subsets of rodent undifferentiated spermatogonia based on clone size (A_single_, A_paired_ and A_aligned_) [25,29]. Stem cell capacity is considered to reside within the A_single_ subset [30,31]. The renewal of A_single_ SSCs occurs by either asymmetric cell division, where only one daughter cell of a dividing SSC retains stemness, or population-level asymmetry, where half of the stem cell divisions produce only stem cells. An alternate “fragmentation model” challenges this view, proposing that the SSC compartment is comprised of the entire undifferentiated spermatogonia pool (A_single_, A_paired_, and A_aligned_ spermatogonia), and these clones are interconvertible and essentially equipotent [27,28]. This model is consistent with the fragmentation proposed by Clermont’s A_0_/A_1_ model and supported by the results of in vivo live imaging studies using reporter mice, which appear to show the separation of clones. This model has been criticized for a lack of definitive identification of the intercellular cytoplasmic bridges defining clones and a failure to confirm cell fate after fragmentation, and thus, remains controversial. Lastly, the “revised A_single_ model” was proposed based on experiments using transgenic *Id4*-eGFP reporter mice, in which undifferentiated spermatogonia were sorted based on the intensity of eGFP epifluorescence into *Id4*-eGFP^Bright^ (SSC-enriched) or *Id4*-eGFP^Dim^ (SSC-depleted) subsets, which have more and less regenerative capacity upon transplantation, respectively [32,33,34]. Given the observation of phenotypic and functional heterogeneity within the *Id4*-eGFP^Bright^ population, which are exclusively arranged as A_single_ spermatogonia, this model holds that a subset of A_single_ are self-renewing SSCs (SSC_ultimate_), while the remainder are in an intermediate state (SSC_transitory_), poised to transition to a progenitor state and likely only contribute to the SSC pool upon perturbations of steady-state conditions [34].

Primate testes contain two subtypes of undifferentiated spermatogonia, A_dark_ and A_pale_, identified based on the differences in their nuclear architecture and staining intensity with hematoxylin [24,35,36]. Initially, Clermont’s group proposed a linear model in which A_dark_ SSCs produce A_pale_ progenitor spermatogonia, which give rise to differentiating B-type spermatogonia [36]. Subsequently, this model was modified based on the observation in Vervet monkeys (*Chlorocebus pygerythrus*) that A_dark_ spermatogonia did not label with a pulse of ^3^H-thymidine, indicating a failure to go through the S-phase and self-renew, while roughly 1/3 of A_pale_ were labeled [37]. Moreover, A_pale_ were cleared following cytotoxic insult, but A_dark_ were not and subsequently began proliferating and regenerated spermatogenesis [37]. Consequently, both A_dark_ and A_pale_ spermatogonia were considered to have stem cell capacity with A_dark_ taking the role of “reserve” stem cells, which only serve as a regenerative backup, while A_pale_ spermatogonia are considered “active” stem cells, which sustain ongoing spermatogenesis in a steady-state [35,38]. Alternatively, others have posited that the low mitotic index of A_dark_ more closely reflects the expected behavior of SSC, while the high turnover of A_pale_ reflects their activity as transit-amplifying progenitor spermatogonia that are actively proliferating to increase the total number of germ cells [39]. More recently, based on molecular phenotyping in nonhuman primates, it has been suggested that A_dark_ and A_pale_ both comprise the SSC pool, but are simply in different phases of the cell cycle [40,41]. Regardless, the lack of tools to functionally and quantitatively measure SSC activity in primate species has precluded a definitive identification of the primate SSC pool.

Recently, single-cell mRNA profiling studies of immature and adult human testicular cells have begun to address the challenge of identifying human SSCs by facilitating subclassification of undifferentiated spermatogonia and defining the molecular signatures for these subsets [33,42,43,44,45,46,47,48]. In human testes, spermatogonia have been classified into undifferentiated (*UTF1+*) and differentiating spermatogonia (*KIT+, MKI67+*). Undifferentiated spermatogonia have been further subdivided into at least four distinct groups with varied levels of key markers including *FGFR3, GFRA1, NANOS2, NANOS3*, *PIWIL4, TSPAN33*, and *UTF1,* highlighting the phenotypic heterogeneity within the undifferentiated spermatogonial compartment. There also appears to be a gradual transition in transcriptome states rather than binary on/off programs [33,44,46,49]. Despite these reports, functional evidence of human SSC capacity is not available to support SSC identity in any of these studies [33,50].

To reduce the uncertainty about the identity of prepubertal human SSCs and understand their developmental relationship with more advanced germ cells, we hypothesized that a comparative analysis of germ cells from the testes of prepubertal nonhuman primates (baboon and rhesus macaque), humans, and mice would allow us to define the most highly conserved phenotype of prepubertal human SSCs. To test this hypothesis, we generated testis single-cell transcriptomes from two prepubertal baboons along with two prepubertal rhesus macaques and defined the spermatogonial transcriptome signatures in both species. To identify a conserved primate SSC gene expression signature diagnostic of the immature human SSC population, we compared the nonhuman primate spermatogonia with their prepubertal human [42,43,44] and mouse [33] counterparts. By determining conservation in gene expression profiles across mammals and unique features in primates, we derived a more refined phenotype for SSCs in the prepubertal human testis, identified pathways that are overrepresented uniquely in primates, and confirmed that SSCs are A_dark_ spermatogonia.

## 2. Results

### 2.1. Single-Cell Transcriptomics Identifies Major Germ and Somatic Cell Types in Prepubertal Human, Baboon, and Macaque Testes

**Figure 1:**
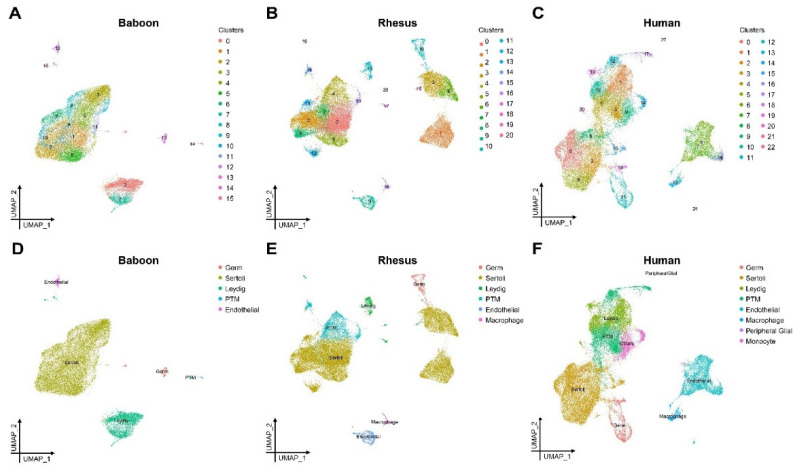
Single-cell RNA-seq analyses of testis cells from prepubertal baboons, rhesus macaques, and humans identify major germ and somatic cell types. (**A**–**C**) UMAP projections of single-cell transcriptomes from the testes of prepubertal (**A**) baboons (*n* = 2), (**B**) rhesus macaques (*n* = 2), and (**C**) humans (*n* = 6). Each dot represents a single cell and is colored according to unbiased cluster annotation (16 baboon clusters, 21 rhesus macaque clusters, and 23 human clusters). (**D**–**F**) UMAP projections showing testicular cells, colored by cell types as determined by differential gene expression, to represent germ and somatic cell types (see Figure S1).

To investigate the cellular diversity during prepubertal testis development in primates, we re-analyzed published human testis single-cell datasets from two newborns [42], two infants [44], and two juveniles [43] (Figure 1A and Table S1), and we performed single-cell RNA-seq (10× Genomics) on unselected testis cells from two prepubertal baboons (newborn and 26 months old; Figure 1B and Table S1) and two prepubertal rhesus monkeys (15 and 20 months old; Figure 1C and Table S1). In addition, we mined existing germ cell transcriptomes from postnatal day (P) 6 mice [33] in order to define the evolutionarily conserved and divergent programs (Figure S2 and Table S1). A total of 92,867 cells (30,752 human, 23,219 baboon, 25,342 rhesus, and 13,554 mouse cells) passed quality control and were used for downstream analyses (Table S1).

Using an unsupervised analysis approach for testis cells from each species, we identified 16, 21, and 23 distinct clusters of cells for baboon, rhesus macaque, and human testis cells, respectively (Figure 1A–C). We observed no bias among the replicates for each species based on cell distributions among the clusters (Figure S1A and Table S2). The clusters were subsequently annotated using established cell-type markers for testicular somatic and germ cells (Figure S1B–D). In the baboon testes, the major cell types consisted of germ cells (*DDX4+*), Sertoli cells (*SOX9*+, *WT1+, DHH+*), Leydig cells (*IGF1+, INSL3*+, *STAR*+), peritubular myoid cells (*ACTA2*+, *MYH11*+), and endothelial cells (*PECAM1*+) (Figure 1D, Figure S1B, Table S2). Similarly, the rhesus testis cells were comprised of germ cells (*DDX4+*), Sertoli cells (*SOX9*+, *DHH+*), Leydig cells (*INSL3*+, *STAR*+), peritubular myoid cells (*ACTA2*+, *MYH11*+) endothelial cells (*PECAM1*+), and macrophages (*AIF1*+) (Figure 1E, Figure S1B, Table S2). From the human testis cells, we found germ cells (*DDX4+*), Sertoli cells (*SOX9*+), Leydig cells (*INSL3*+, *STAR*+, *IGF1+*), peritubular myoid cells (*ACTA2*+, *MYH11*+), endothelial cells (*PECAM1*+), macrophages (*AIF1*+), peripheral glia (*S100B+*, *LGI4+*), and monocytes (*HSPA6+, RND3+, TCMI+*) (Figure 1F, Figure S1B, Table S2). Notably, we found interspecies differences in terms of the cell types present. Specifically, we identified a testicular macrophage population in the human and rhesus testis cells, which was absent from the baboon testis cells. We also noted differences in expression of somatic cell type specific markers, including an absence of *WT1* mRNAs in rhesus Sertoli cells and a lack of *DHH* in human Sertoli cells.

### 2.2. Focused Analysis of Germ Cells Reveals Discrete Spermatogonial Subtypes in Prepubertal Humans

**Figure 2:**
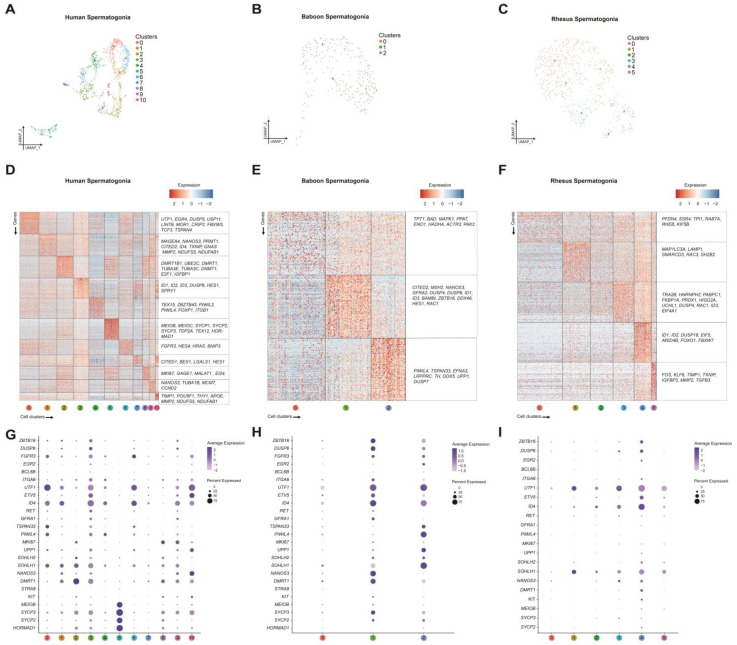
Focused single-cell transcriptome analysis of germ cells from prepubertal human, baboon, and rhesus macaque testes. (**A**–**C**) UMAP plot visualization of germ cells from (**A**) human (*n* = 1451), (**B**) baboon, (*n* = 233) and (**C**) rhesus (*n* = 599) testes colored by unbiased cluster as noted in the legend for each species. (**D**–**F**) Heatmaps show the top 100 significantly differentially expressed genes between each unbiased cell cluster (noted by the numbers at the bottom) and expression of the key markers for each gene cluster (right) for (**D**) human, I baboon, and (**F**) rhesus macaque germ cells. Gene lists can be found in Table S2. (**G**–**I**) Dot plot showing the expression of the selected markers of undifferentiated and differentiated spermatogonia for unbiased clusters in (**G**) human, (**H**) baboon, and (**I**) rhesus macaque datasets used to identify germ cell subsets. The size of the dot represents the percentage of cells within a cell type, while the color encodes the average expression level across all cells within a cell type (blue is high).

To gain a better understanding of prepubertal germ cell heterogeneity in humans, we performed a focused analysis of the 1451 germ cells identified in the human testis cell cluster 11 (Figure 1A). Unsupervised re-clustering of the human germ cells visualized in a UMAP projection revealed 11 unbiased cell clusters (Figure 2A). The identity of the cells within these clusters were scrutinized by examining 5289 genes differentially expressed between all the clusters (Figure 2D and Table S2), as well as expression of genes known to distinguish undifferentiated spermatogonia (*BCL6B, DUSP6, EGR2, ETV5, FGFR3, GFRA1, ID4, ITGA6, PIWIL4, RET, UTF1,* and *ZBZTB16*) from early differentiating spermatogonia *(DMRT1, KIT NANOS3, SOHLH1, SOHLH2, STRA8,* and *UPP1*) and late differentiating spermatogonia that have activated the meiotic gene program (*HORMAD1, MEIOB, SYCP2,* and *SYPC3*) (Figure 2D). The cells in clusters 0, 1, and 6 expressed elevated levels of genes consistent with SSCs, including *FGFR3*, *ID4, PIWIL4, TSPAN33,* and *UTF1* (Figure 2D,G and Table S2). Along with these prototypical SSC genes, enhanced expression of novel genes was also observed in cluster 0 (*DUSP5, EGR4, FBXW5*, *TCF3,* and *TSPAN4*), cluster 1 (*GNAS* and *MMP2*), and cluster 6 (*BNIP3, HES4,* and *HRAS*) (Figure 2G and Table S2). Furthermore, although the cells in cluster 1 exhibited lower levels of *FGFR3* and *UTF1* mRNA, they also had low levels of *DUSP6* and *ZBZTB16*, possibly indicating that these cells are transitioning towards a progenitor state. In addition to expressing markers of undifferentiated spermatogonia *(DUSP6, ETV5, ITGA6,* and *ZBZTB16*), the cells in clusters 3, 4, 7, 9, and 10 were also enriched for progenitor markers (*NANOS3* and *UPP1*), thus representing progenitor spermatogonia (Figure 2D,G and Table S2). In contrast, clusters 2 and 8 were designated as early differentiating spermatogonia based on the elevated expression of *DMRT1, KIT, MKI67*, and *SOHLHL2* (Figure 2D,G and Table S2). Lastly, the cells in cluster 5 were considered late differentiating spermatogonia because their distinct transcriptome featured elevated levels of meiotic genes known to be activated during spermatogonial differentiation, including *MEIOB, SYCP2, SYCP3*, and *HORMAD1* (Figure 2D,G and Table S2) [33].

### 2.3. Transcriptomic Characterization of Prepubertal Baboon Germ Cells

Among baboon testis cells, cluster 11, comprising a total of 233 cells, expressed the germ cell marker *DDX4* (Figure 1B,E). When reanalyzed in isolation, the cells in this cluster further resolved into three cell clusters (clusters 0–2), which were distinguished based on 1256 genes differentially expressed between all the clusters (Figure 2B,E and Table S2). However, unlike human germ cell clusters, which appeared as distinct groups on the UMAP projection, the baboon germ cells largely appeared to cluster together. The cell in Cluster 0 expressed lower levels of both undifferentiated (*ETV5, PIWIL4,* and *TSPAN33*) and differentiated (*DMRT1* and *SOHLH1*) spermatogonial markers (Figure 2E,H). Both clusters 1 and 2 displayed similar patterns of elevated gene expression for the undifferentiated spermatogonial markers *FGFR3, ID4,* and *UTF1* (Figure 2H). Cluster 1 uniquely expressed the undifferentiated spermatogonial markers *DUSP6, ETV5, GFRA1, RET, TSPAN33,* and *ZBZTB16*, whereas cluster 2 uniquely expressed *EGR2* and *PIWIL4* (Figure 2H). In addition, there was also expression of differentiating spermatogonial markers in cluster 1 (*DMRT1*, *KIT,* and *NANOS3*) and cluster 2 (*SOHLH1*) (Figure 2H). Moreover, meiotic markers *SYCP2* and *SYCP3* were expressed at a higher level in cluster 1 compared to cluster 2 (Figure 2H), suggesting some of these cells are more advanced. Since the baboon germ cell clusters expressed varying levels of the markers indicative of both undifferentiated and differentiating spermatogonia, it was not possible to annotate the cell types like we did in humans (Figure 2E,H).

### 2.4. Transcriptomic Characterization of Prepubertal Rhesus Macaque Germ Cells

Using the same approach as we did for human and baboon germ cells, we identified a total of 599 rhesus germ cells based on the expression of *DDX4* and performed iterative re-clustering of these cells, yielding six spermatogonial clusters (clusters 0–5) distinguished based on 1382 differentially expressed genes between all clusters (Figure 2C,F and Table S2). Similar to baboons, though, rhesus germ cells clustered together and did not exhibit distinct spermatogonial groups based on gene expression (Figure 2F,I). Specifically, cluster 0 mostly lacked expression of undifferentiated and differentiating marker genes, with few cells expressing *NANOS3* (Figure 2I). Clusters 1–5 were characterized by varying levels of *ID4* and *SOHLH1* expression. Interestingly, Clusters 1, 2, and 5 largely lacked expression of any other undifferentiated and differentiating marker genes. In contrast, cluster 4 had elevated expression of markers of both undifferentiated (*ZBZTB16, DUSP6, EGR2, ETV5,* and *ID4*) and differentiating spermatogonia (*DMRT1, KIT, NANOS3,* and *SOHLH1*). We also observed species-specific features, including the absence of *FGFR3, PIWIL4*, and *TSPAN33* expression in rhesus spermatogonia. Furthermore, the expression of meiosis-related genes (*HORMAD*, *MEIOB, SYCP2*, and *SYCP3*) was essentially absent in rhesus spermatogonia, indicating the absence of late differentiating spermatogonia. Thus, rhesus spermatogonia were clustered together and exhibited gradual transitions in marker expression, making it difficult to distinguish spermatogonial subtypes. (Figure 2F,I).

### 2.5. Cross-Species Comparison of Spermatogonia Reveals Conserved and Divergent Features in Prepubertal Primate and Rodent Spermatogonia

**Figure 3:**
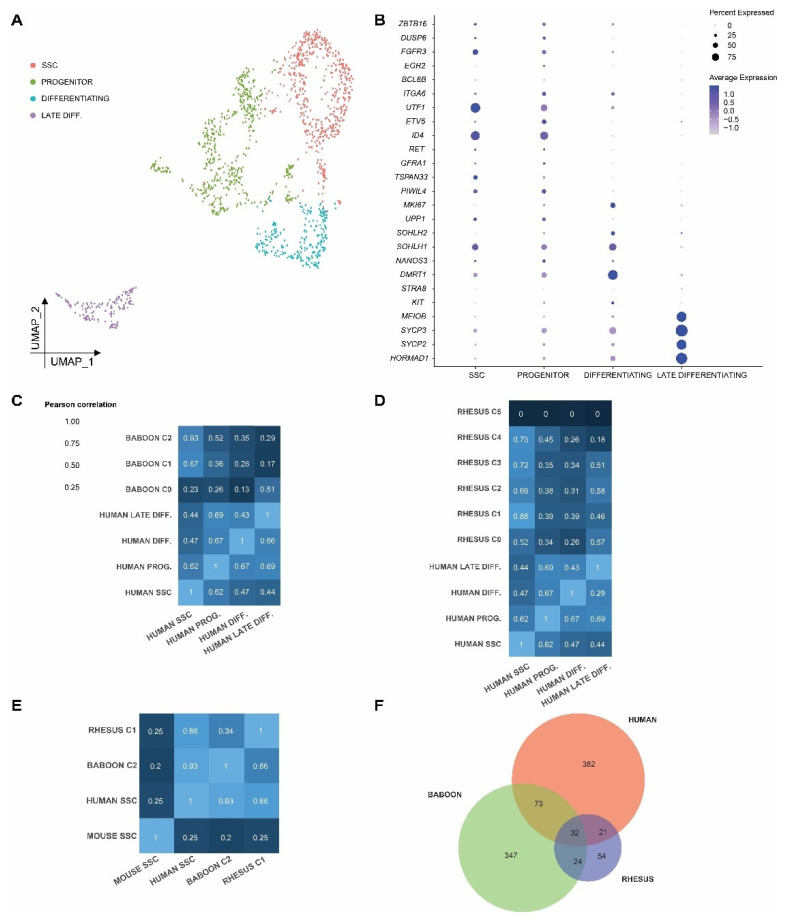
Cross-species comparison of putative human SSCs revealed analogous cell types in baboon and rhesus and unique markers of primate SSCs. (**A**) UMAP plot colored by cell type annotation represents human germ cells subsets (*n* = 1451). (**B**) Dot plot shows the expression of selected markers for each germ cell subset seen in the UMAP plot. The size of the dot represents the percentage of cells within a cell type, while the color encodes the average expression level across all cells within a cell type (blue is high). (**C**–**D**) Correlation heatmaps show the comparison of the human SSC cluster with (**C**) baboon or (**D**) rhesus unbiased spermatogonial clusters, colored based on the Pearson correlation coefficient. (**E**) Correlation heatmap shows the comparison of putative SSC clusters from mouse, human, baboon, and rhesus germ cell datasets, colored based on the Pearson correlation coefficient. (**F**) Venn diagram shows the comparison of genes differentially expressed in the human SSC cluster (*n* = 508) with genes differentially expressed in the putative SSC clusters in baboon and rhesus. Thirty-two genes were conserved in humans, baboons, and rhesus macaques and represent markers of putative SSCs.

To align the undifferentiated spermatogonial clusters/subsets across these three primate species, we first performed a joint analysis of the germ cells from each species by comparing primate germ cell subtypes with transplant-validated mouse SSCs (Figure S3A–C). For this purpose, we used 1-1-1-1 orthologous genes (*n* = 9068) to merge the gene expression data for 1451 human, 233 baboon, 599 rhesus, and 10,012 mouse germ cells [33]. To identify analogous spermatogonial subsets across the species, we defined the whole-transcriptome correlation from each of the three primate species to those from mice (Figure S3A). Surprisingly, cells in human cluster 5, which expressed transcriptomes consistent with late differentiating spermatogonia (see Figure 3), were most highly correlated with mouse SSCs (Pearson coefficient = 0.34) compared with the remaining human germ cell clusters (Pearson correlation coefficients ranging 0.01–0.33; Figure S3A). Among all human germ cells clusters, cluster 5 was also the most highly correlated with mouse progenitors (Pearson correlation coefficient = 0.40; Figure 3A) and mouse differentiating spermatogonia (Pearson correlation coefficient = 0.60; Figure S3A). Identical comparisons of baboon germ cell clusters with mouse spermatogonial subtypes revealed that baboon cluster 0 was most similar to mouse SSCs (Pearson correlation coefficient = 0.34; Figure S3C), while cluster 2 was the most highly correlated with both mouse progenitors (Pearson correlation coefficient = 0.33; Figure S3C) and mouse differentiating spermatogonia (Pearson correlation coefficient = 0.38; Figure S3C). Likewise, rhesus cluster 5 was the most highly correlated with mouse SSC (Pearson correlation coefficient = 0.35; Figure S3D), mouse progenitors (Pearson correlation coefficient = 0.36; Figure S3D) and mouse differentiating spermatogonia (Pearson correlation coefficient = 0.40; Figure S3D). Thus, although functionally informed, a comparison of the mouse spermatogonial subtypes to the unbiased groups of primate undifferentiated spermatogonia indicates that the degree of transcriptome similarity between primate and rodent germ cells is relatively low and may not predict cell type identity in primates, likely due to evolutionary distance.

Therefore, we assigned human cell identity based on the relative abundance of mRNA for proposed and established markers of undifferentiated spermatogonia (*FGFR3, ID4, PIWIL4, TPSAN33,* and *UTF1*) versus progenitor, differentiating the spermatogonia and meiotic markers (Figure 2H). In addition to the use of these markers, global differential gene expression analysis between the unbiased human germ cell clusters facilitated assignment of cell type identities, SSC (Human germ cell clusters C0, C1, and C6), progenitor (Human C3, C4, C7, C9, and C10), differentiating (Human C2 and C8), and late differentiating (Human C5) cells, which are represented as a UMAP projection (Figure 2D, Figure 3A and Table S1). A comparison of the human germ cells bearing these cell type assignments with mouse spermatogonia revealed that the mouse SSCs were still most highly correlated with human differentiating spermatogonia (Pearson correlation coefficient = 0.38; Figure S3B). In contrast, human progenitors were most correlated with mouse progenitors and human late differentiating spermatogonia were most correlated with mouse differentiating spermatogonia, indicating that more advanced germ cells share a more similar transcriptome across species (Figure S3B).

To identify the analogous spermatogonial subsets in baboons and rhesus macaques, we compared those cells identified as prepubertal human SSCs with clusters of baboon and rhesus macaque germ cells. We found that the cells in baboon cluster 2 were the most highly correlated with human SSCs (Pearson correlation coefficient = 0.93; Figure 3C), while the cells in rhesus cluster 1 were the most correlated with human SSCs (Pearson correlation coefficient = 0.86; Figure 3D). Human progenitors were most highly correlated with baboon cluster 2 (Pearson correlation coefficient = 0.52; Figure 3C) and rhesus macaque cluster 4 (Pearson correlation coefficient = 0.45; Figure 3D). Human differentiating spermatogonia were most highly correlated with baboon cluster 2 (Pearson correlation coefficient = 0.35; Figure 3C) and rhesus macaque cluster 4 (Pearson correlation coefficient = 0.45; Figure 3D). In addition, human late differentiating spermatogonia were most highly correlated with baboon cluster 0 (Pearson correlation coefficient = 0.51; Figure 3C) and rhesus macaque cluster 2 (Pearson correlation coefficient = 0.58; Figure 3D). A subsequent comparison of these putative primate SSCs with transplant-validated mouse SSCs demonstrated a low correlation with human (correlation coefficient = 0.25), baboon (correlation coefficient = 0.20), and rhesus (correlation coefficient = 0.24) SSCs (Figure 3E). Reciprocally, primate SSCs were much more highly correlated to each other, which likely reflects the evolutionary distance between primates and rodents (pairwise correlation coefficients 0.86 and 0.93 Figure 3E).

Having identified the SSCs in each of the primate species, we next sought to identify the conserved components of the primate SSC gene expression signature (i.e., markers of primate SSCs). For this purpose, differential expression analysis was performed between human SSCs, progenitors, and differentiating spermatogonia, revealing 508 genes with higher expression in human SSC compared to other human spermatogonial subtypes. Similarly, putative baboon SSCs (Baboon C2) uniquely expressed higher levels of 347 genes and Rhesus SSCs (Rhesus C1) expressed higher levels of 54 genes (Table S2). Thirty-two of these genes were markers of SSCs in all three species (Figure 3F and Table S2). Among these genes, only *PMAIP1* and *RPL22L1* were also conserved in mouse SSCs. Next, we determined the expression of these 32 genes in publicly available datasets using the Mammalian Reproductive Genetics Database (MRGDv2; https://orit.research.bcm.edu/MRGDv2, accessed on 23 December 2022) (Figure S4) [51]. Congruent with our data, a majority of these 32 primate SSC markers were expressed in SSEA4+ human spermatogonia (undifferentiated), but absent from KIT+ differentiating spermatogonia (Figure S4). However, the majority of the primate SSC markers were expressed by both mouse SSCs (ID4 + high) and progenitors (ID4 + low) (Figure S4). In contrast to primates, mouse spermatogonia lacked expression of a number of these markers (*Sh2b2, Rpl32*) (Figure S4).

**Figure 4:**
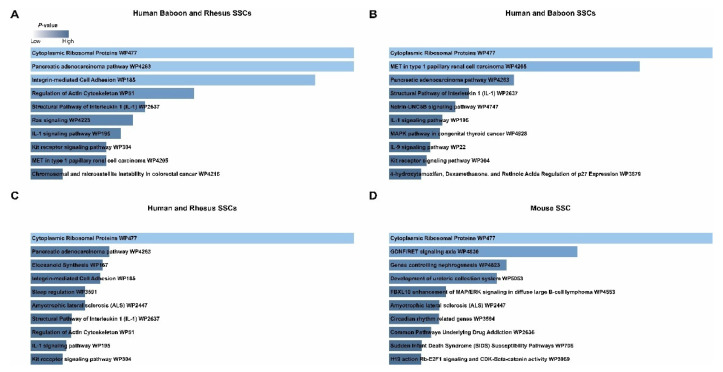
Pathways analyses reveal unique biological pathways among conserved primate SSC markers. Pathway analysis of the (**A**) 32 markers of human, baboon, and rhesus SSCs, (**B**) 105 markers of human and baboon SSCs, (**C**) 53 markers of human and rhesus SSCs, and (**D**) 127 markers of mouse SSCs. The lengths of the horizontal bars and their color represent *p*-values according to the scale.

Gene ontology analyses indicated that the 32 conserved primate SSC markers were enriched for genes involved in “Cytoplasmic Ribosomal Proteins” (*RPL32, RPL18, RPS12;* Figure 4A and Table S2). This same pathway was overrepresented in conserved markers between both human and baboon SSCs (*RPL32, RPL11, RPSA, RPL18, RPS12*; Figure 4B and Table S2), as well as human and rhesus SSCs (*RPS4X, RPS9, RPL32, RPL18, RPS12*; Figure 4C). Like primate SSCs, “Cytoplasmic Ribosomal Proteins” (*Rpl4, Rps4x, Rps28, Rps27, Rps29, Rpl37, Rpl39, Uba52;* Figure 4D and Table S2) were overrepresented among mouse SSC markers, pointing to translational control as a common feature of mammalian SSCs. However, although the same pathway was identified in the GO analysis of primate SSCs, the specific marker genes were different in primates and rodents, indicating possible differences in the precise mechanisms that regulate translation.

Three genes (*MAP2K2, RAC3,* and *PAK4*) that were conserved in the SSCs of all three primates were also involved in numerous pathways, including “Pancreatic adenocarcinoma pathway”, “Integrin-mediated Cell Adhesion”, “Regulation of Actin Cytoskeleton”, and “Ras signaling” (Figure 4A–C). These pathways and genes were absent from the mouse SSC markers, indicating a species-specific role in SSC biology among primates. The genes involved in IL-1 signaling pathway (*MAP2K2* and *TOLLIP;* Figure 4A and Table S2) were also enriched in the SSCs from all three primate species. Curiously, the genes annotated as involved in KIT receptor tyrosine signaling (*MAP2K2* and *SH2B2;* Figure 4A and Table S2), ordinarily associated with spermatogonial differentiation, were also observed in primate SSCs despite the lack of *KIT* expression (Figure 2H,I and Figure 3B), which may suggest unique primate-specific signaling cascades that converge at these factors.

**Figure 5:**
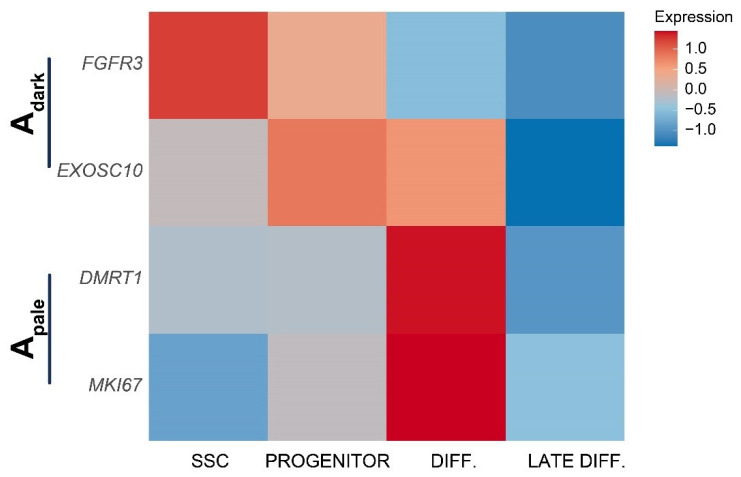
Marker expression of A_dark_ and A_pale_ spermatogonia in prepubertal human spermatogonia. Heatmap shows mRNA expression of A_dark_ (*EXOSC10, FGFR3*) and A_pale_ (*MKI67, DMRT1*) markers in human SSCs, progenitors, differentiating spermatogonia, and late differentiating spermatogonia (red is high, blue is low).

To use our data, we sought to relate our primate SSC signatures with the histological definitions of human A_dark_ and A_pale_ spermatogonia based on nuclear staining intensity with hematoxylin. We examined our data for markers that are known to discriminate A_dark_ and A_pale_ by immunostaining. Previous studies demonstrated that FGFR3 and EXOSC10 are restricted to rarefaction zone-containing A_dark_ spermatogonia, while MKI67 and DMRT1 were absent from these cells [50,52]. We found that *FGFR3* was expressed by SSCs and progenitors, *EXOSC10* by progenitors and differentiating spermatogonia, and both *DMRT1* and *MKI67* were restricted to differentiating spermatogonia (Figure 5) [53]. These data indicate that the SSCs and progenitors are all A_dark_, while cells that have initiated differentiation are A_pale_ (Figure 5).

## 3. Discussion

The field of spermatogonial biology has struggled to propagate primate SSCs in culture, which would enable their routine use in experimentation, as well as exploiting their therapeutic potential to restore spermatogenesis. Although attempts at in vitro human SSC culture have been made, a robust culture system for human SSCs is yet to be developed [12,13,18,54]. Indeed, supporting evidence proving spermatogonial identity (let alone SSCs) is weak or circumstantial and none of the reported conditions have been robustly repeated by external groups. Thus, the field still lacks the ability to maintain primate (including human) SSCs in culture. By refining the molecular characteristics of SSCs in humans, baboons, and rhesus macaques, we have identified potential culture considerations that may finally permit the expansion of human SSCs.

The major objective of our study was to refine the identity and phenotype of SSCs in primate species. To this end, we employed a single-cell RNA-seq to define the germ and somatic cell types in prepubertal human, baboon, and rhesus testes, and focused our analyses on determining the degree of gene expression conservation among putative primate SSCs relative to functionally defined cells in mice. Specifically, we found 32 genes conserved and uniquely enriched in primate SSCs. Three genes in particular (*MAP2K2*, *PAK4*, and *RAC3*) exhibited a conserved expression pattern in the SSCs from all three primates and are also involved in integrin-mediated cell adhesion, the regulation of the actin cytoskeleton, and cell migration. Among these genes, MAP2K2, or mitogen-activated protein kinase kinase 2, is already known to be involved in cell fate determination, cell growth, and differentiation [55]. PAK4 or p21 (RAC1) activated kinase 4 is required for SSC homing and transmigration through the blood-testis barrier in mice [56]. RAC3 is a small GTPase that interacts with the integrin binding protein CIB1 (calcium and integrin binding 1) to promote cell adhesion [57]. In addition, the genes implicated in the positive regulation of cell migration (CIB1 and PRR5) were enriched in the primate SSCs. Overall, these results suggest that genes involved in cell adhesion, regulation of the actin cytoskeleton, and cell migration may play an outsized role in SSC function in primates. In contrast to primates, genes enriched uniquely in mouse SSCs were involved in the GDNF/RET signaling axis (*Gfra1*, *Ret*, *Foxc2*, and *Lhx1*), which is known to be important for mouse SSC self-renewal. In mice, the addition of GDNF and FGF2 to culture medium is essential for long-term propagation of SSCs [58]. Absence of the GDNF/RET signaling axis in primate SSCs may explain why attempts to culture and expand human SSCs using this factor have not been fruitful. Instead, unique features of primate SSCs that are not prominent in rodents may need to be investigated in order to propagate human SSCs in vitro.

The genes implicated in ribosome biosynthesis and the regulation of translation were enriched in the SSCs from all four species of mammals. Specifically, genes encoding structural components of ribosomes and translational regulation were observed. These data raise the interesting concept that post-transcriptional gene regulation is involved in establishing the stem cell state in mammals. Decades of work have set the clear precedent for the concept of post-transcriptional regulation of germ cell development in invertebrates, lower vertebrates (*C. elegans*, *Drosophila*, *Xenopus*) [59,60,61], and in mammals [62,63]. However, the precise role for translational regulation in maintenance of the SSC state has never been firmly determined. Future studies may leverage this knowledge to promote enhanced translation of specific messages and prevent translation of others in hopes of driving enhanced SSC renewal.

Importantly, the correlation of human spermatogonial single-cell transcriptomes (SSC, progenitors, differentiating, and late differentiating) with markers of A_dark_ (expressing *EXOSC10* and *FGFR3*) and A_pale_ (expressing *MKI67* and *DMRT1*) spermatogonia provided a prediction of the likely histological phenotype of molecularly-defined SSCs. These data indicated that human SSCs are a subset of A_dark_ and lack *MKI67* expression, consistent with the notion of a slow-cycling stem cell population [35,37,38]. Progenitor spermatogonia were also found in the A_dark_ population, arguing for a single pool of SSCs that is comprised entirely of A_dark_ spermatogonia. Intriguingly, the A_pale_ spermatogonial population appear to consist of cells that are proliferative and initiate differentiating, which is incongruent with the designation of A_pale_ as active SSCs, but supportive of their role as a transit-amplifying progenitor poised to differentiate.

Since the generation of single-cell suspensions for scRNA-seq necessitates the dissociation of the three-dimensional testis tissue architecture, we are unable to directly associate the SSC transcriptome phenotypes with their native context within seminiferous tubules. Previous studies have shown that the self-renewal and differentiation of SSCs is influenced by their microenvironment [64,65]. Therefore, spatial transcriptomics could potentially overcome this key limitation of traditional single-cell profiling and provide crucial insights into the regulatory mechanisms of spermatogenesis. Using Slide-seq2, Chen et al. performed spatial analysis of adult human and mouse testis using ~29 K beads to capture transcripts. This analysis revealed differences in the spermatogonial compartments and the cellular compositions of the spermatogonial microenvironment between humans and mice [66]. In addition, the study described spatially resolved expression patterns. For instance, *Smcp* expression was enriched in round spermatids near the tubule lumen, while *Lyar* expression was elevated in the spermatocytes near the basement membrane. Future studies may overcome the relatively low resolution of this method, which limits investigation of cell–cell interactions, such as ligand–receptor relationships [66].

Precise analysis of expression patterns of the ligand receptor in SSCs and their supporting somatic cells has been possible using scRNA-Seq and led to predictions of the interactions between distinct cell types. Several studies have used this approach to suggest that FGF, GDNF, KIT, retinoic acid, and activin/inhibin signaling as the most relevant to human spermatogenesis [42,43,45,49]. Our results fail to corroborate many of these predictions, and instead suggest that prototypical examples, like GDNF (GFRA1 and RET) and FGF (FGFR3), are false positives, likely the result of flawed cell type designation. Our data do support the potential for activin/inhibin signaling insofar as we identified expression of ACVR1/2 receptors in primate SSCs, suggesting this pathway may be important for the SSC state. Future studies may help corroborate ligand–receptor analysis using protein data.

In conclusion, scRNA-seq identified SSCs in humans, macaques, and baboons highlighted a surprising phenotypic discordance between primate SSCs and functionally defined mouse SSCs. These results may explain the difficult adapting conditions for the propagation of mouse SSCs to primates. Importantly, primate SSCs were enriched for transcripts encoding components and regulators of the actin cytoskeleton and regulation of cell-adhesion, which may be the missing pieces necessary to devise a robust protocol for in vitro propagation of human SSCs. Addressing a nearly half-century debate, our data confirm that SSCs are a subset of A_dark_ spermatogonia, putting to rest the concept of a dual stem cell system in primate testes. Taken together, these results refine the identity of prepubertal human SSCs and elucidate molecular features that may be leveraged for their use in the clinic.

## 4. Materials and Methods

### 4.1. Animals

The testicular tissues were recovered at necropsy at the Southwest National Primate Research Center (SNPRC, Texas Biomedical Research Institute) as biomaterials collections from two immature baboons (postnatal day 0 and 26 months) and two rhesus macaques (15 months and 20 months) after they had been euthanized for another purpose. Therefore, the research conducted with these tissues was not considered to be animal research.

### 4.2. Single-Cell Suspension Preparation

The donor cells were recovered from olive baboon and rhesus macaque testes using a two-step enzymatic digestion approach as previously described [67]. Briefly, testicular parenchyma was digested with 1 mg/mL Collagenase Type IV (Worthington Biochemicals) for 2–3 min at 37 °C, washed with Hank’s Buffered Salt Solution (HBSS) to remove the interstitial cells, digested with 0.25% trypsin/EDTA containing 1.4 mg/mL DNase I (Sigma) for 7–9 min at 37 °C, and quenched with 10% FBS. The testis cell suspensions were filtered through a nylon mesh to generate a single-cell suspension. The cell yield was determined by counting the cells using a hemocytometer and viability was assessed by trypan blue exclusion.

### 4.3. Single-Cell RNA-Sequencing of Baboon and Macaque Testis Cells

Single-cell transcriptomes were generated using the 10× Genomics Chromium Cell Gene Expression kit (v2 chemistry) per manufacturer recommendations [68] at the UTSA Genomics Core as previously described [33]. For each replicate, we targeted a collection of 5000 single-cell gel bead emulsions (GEMs) containing single cells and generated libraries as per manufacturer recommendations [68]. The libraries were sequenced at the Genome Sequencing Facility (GSF) at Greehey Children’s Cancer Research Institute at UT Health San Antonio (UTHSA) on a HiSeq2500 (Illumina) instrument. The 10× Genomics Single-cell 3’ libraries were prepared according to manufacturer recommendations (Single Cell 3’ v3 chemistry for 37979, Single Cell 3’ v2 chemistry for all others). Illumina base-calling and demultiplexing was performed with BCL-2-Fastq.

The trimmed FASTQ files were generated using the Cell Ranger v3.1.0 (10× Genomics) mkfastq command using the default parameters. The alignment of the trimmed reads, filtering, and UMI counting were performed using the CellRanger v3.1.0 count function using mouse GRCm38 (mm10), human GRCh38 (hg38), *Papio anubis* (olive baboon) Panubis1.0 (Ensemble GCA_008728515.1), and *Macaca mulatta* Mmul_10 (Ensemble GCA_003339765.3) references. For each replicate, a single gene-barcode matrix file (MTX) was produced. Each sparse matrix is stored in Market Exchange Format (MEX), which contains TSV files with features (genes) and barcode sequences corresponding to the row and column indices, respectively. Our baboon and rhesus macaque scRNA-seq datasets are publicly available (GSE222105). 

### 4.4. Quality Control, Pre-Processing, and Integration of Gene Expression Datasets

We compared the nonhuman primate datasets generated from these libraries we produced to the published datasets representing testis cells from prepubertal humans at PD2 and PD7 (GSE124263) [42], 12 mo, 13 mo (GSE120508) [44], and 7 yo, 11 yo GSE134144 [43], along with previously published single-cell datasets from postnatal day 6 (P6) mouse testes [33] (see Table S1). The gene expression matrices for each dataset were imported into Seurat (v3.2.3) [69] using the Read10X function and merged by species using the merge function. The merged datasets were subsequently filtered for cells expressing ≥ 200 detected genes and genes expressed in 3 ≥ cells. SCTransform normalization was performed on each of the merged datasets using the command sctransform, which performs normalization, variance stabilization, and selection of variable genes in one step. We subsequently filtered low-quality cells exhibiting >20% reads mapping to mitochondrial genes using the vars.to.regress argument. Integration was performed by calculating the Pearson residuals and identifying anchors. Cell clustering and visualization was performed using the FindNeighbors, FindClusters, and RunUMAP functions, using a resolution of 0.5 based on the principal components selected using the JackStraw function. The FindMarkers function was used to run differential expression analysis for clusters or using cell type annotations. For the iterate clustering of germ cells, the subset function was used to extract the germ cell clusters.

### 4.5. Cross-Species Correlation Analysis of Germ Cells

Orthologues were retrieved from Ensembl Biomart (Ensembl Gene Version 94, Sept 2021). The pairwise orthologues for human vs. baboon (*n* = 18,131), human vs. rhesus (*n* = 21,616), and human vs. mouse (*n* = 20,390) were retrieved separately. These pairwise orthologues were used to subset each of the baboon, rhesus, and mice datasets separately in Seurat using the human Ensembl gene ID as reference. Subsequently, a merged dataset of all 4 species was produced using the merge function in Seurat, with expression data for one-to-one-to-one-to-one orthologues only for further analysis. Subsequently, the correlation coefficients were calculated between the clusters or cell types using the AverageExpression function in Seurat and the R package tidyr (v1.1.4) [70]. The R package gpplot2 (v3.3.5) was used to visualize the correlation heatmaps [71]. Gene ontology enrichment analysis was performed using EnricherR using the pathway database Wikipathways [72,73].

## Data Availability

All newly generated data reported in this study has been deposited in Gene expression Omnibus (GEO) database under the accession number GSE222105.

## Supplementary Materials

The following supporting information can be downloaded at: https://www.mdpi.com/article/10.3390/ijms24054755/s1.
