# Supplementary material for: Conserved Transcriptome Features Define Prepubertal Primate Spermatogonial Stem Cells as Adark Spermatogonia and Identify Unique Regulators
Source: Int J Mol Sci. 2023 Mar 1;24(5):4755. doi: 10.3390/ijms24054755 (PMC10002546; doi:10.3390/ijms24054755)
Supplement: Supplementary file 1 [file ijms-24-04755-s001.zip › ijms-2223567-supplementary.pdf]

Table S1. Prepubertal testis single-cell RNA-seq datasets.

	Guo et al., 2018 [44]	Sohni et al., 2019 [42]	Guo et al., 2020 [43]	Hermann et al., 2018 [33]	This Study	This Study
**Age**	(infant) 12 and 13 months	(newborn) 2 and 7 days	(juvenile) 7 and 11 years	Postnatal day (P) 6	15 and 20 months	Postnatal day 0, 26 months
# **Testis cells**	2245<br>Infant1_1- 568<br>Infant1_2- 467<br>Infant2_1- 597<br>Infant2_2- 613	24,780<br>D2_I—2353<br>D2_T—6490<br>D7_I—11,767<br>D7_T—4170	3727<br>11 yo_rep1—1069<br>11 yo_rep2—700<br>7 yo_rep1—991<br>7 yo_rep2—967	13,554<br>Bright—5552<br>Dim – 4391<br>Unselected—3611	23,219<br>B36506A—9306<br>B36506B—5921<br>B37979—7992	25,342<br>R34403—9935<br>R37136A—8253<br>R37136B—7154
**Mean Reads per Cell**	Infant1_1-79,287<br>Infant1_2-91,489<br>Infant2_1-131,936<br>Infant2_2-137,661	D2_I—31,753<br>D2_T—21,702<br>D7_I—21,792<br>D7_T—25,900	11 yo_rep1—166,257<br>11 yo_rep2—44,427<br>7 yo_rep1—86,716<br>7 yo_rep2—129,258	Bright—108,179<br>Dim—117,390<br>Unselected—160,375	B36506A—62,329<br>B36506B—73,549<br>B37979—51,717	R34403—70,364<br>R37136A—55,596<br>R37136B—81,417
**Replication**	Two technical replicates from each of two individuals	One technical replicate of ITGA6-enriched (I) or unsorted cells (T) from each of two individuals	Two technical replicates from each of two individuals	One technical replicate per sample derived from multiple pups in one litter	Two technical replicates from B36506, one technical replicate from B37979	Two technical replicates from R37136, one technical replicate from R34403
**Accession #**	GSE120508	GSE124263	GSE134144	GSE108970	GSE222105	GSE222105

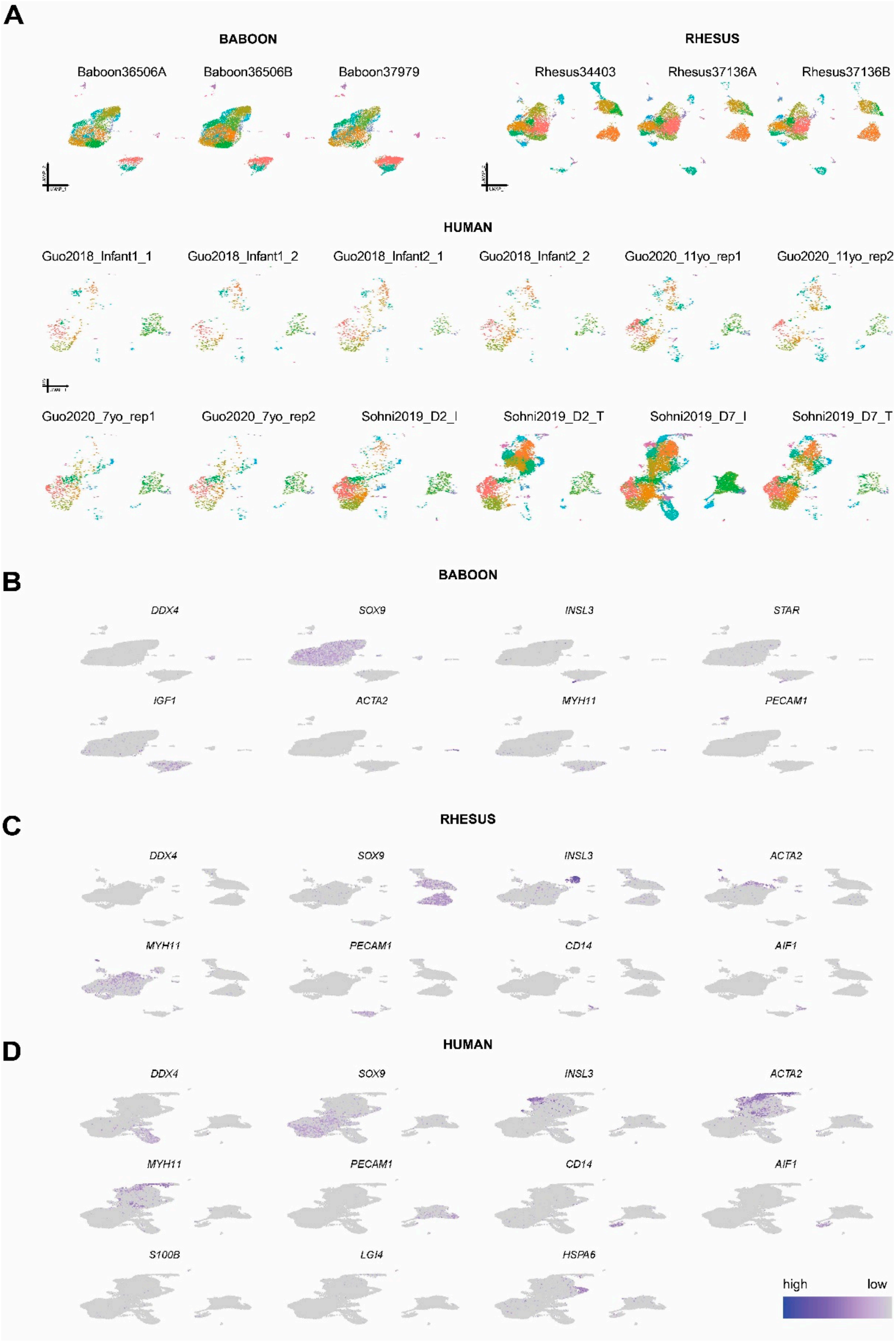

Figure S1. Profiling of single-cell transcriptomes of prepubertal human, baboon, and macaque testicular cells. UMAP plot visualization of the testicular cells from (**A**) baboon, rhesus macaque, and human displayed by replicates (same data as Figure 1). Each dot represents a single cell, which is colored by cluster annotations. (**B–D**) UMAP plots showing the testicular cells from (**B**) baboon, (**C**) rhesus macaque, and (**D**) human testes, which are color-coded to show the expression pattern of the selected gene markers specific for germ and somatic cell lineages.

Table S2. Differentially expressed genes and pathway analyses related to Figures 1–3. An Excel spreadsheet with multiple tabs relating to Figures 1–3. Each tab is labeled according to the relevant figure panel.

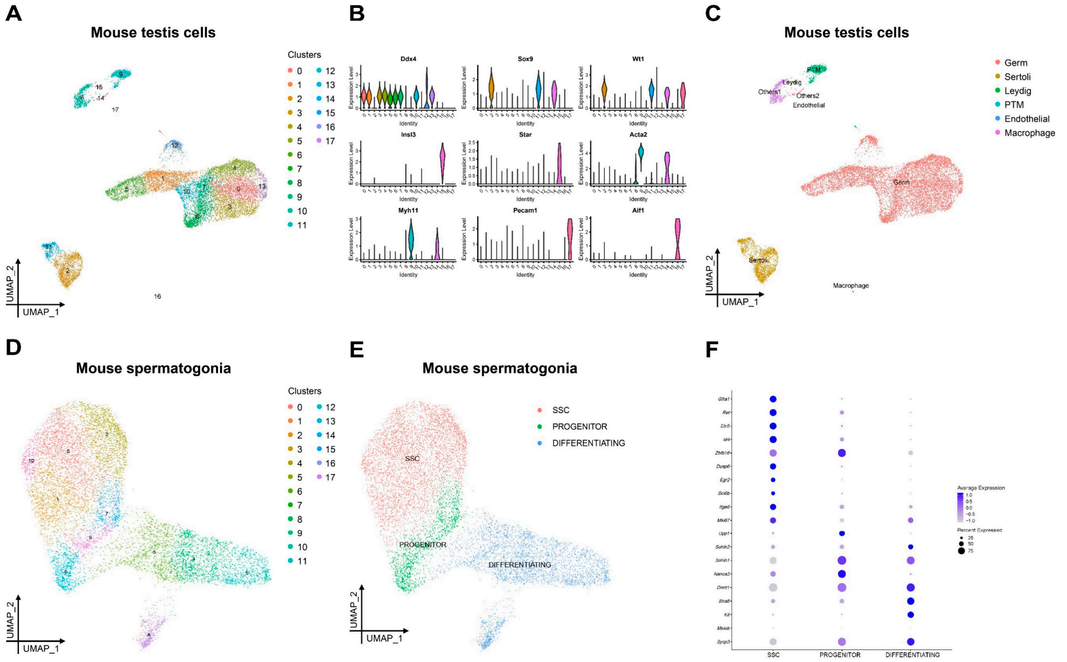

Figure S2. Single-cell transcriptome analysis of testis cells from postnatal day 6 (P6) mouse. (A and C) UMAP plot visualization of single-cell transcriptomes from P6 mouse testis (*n*=13,554) colored by **(A)** unbiased cluster annotation (*n*=18) and **(C)** colored by cell type. **(B)** Violin plots of mouse testes cells which are color-coded according to cluster annotations showing the expression of selected gene markers specific for germ and somatic cell lineages. **(D-E)** UMAP projections showing re-clustering of mouse germ cells colored by **(D)** unbiased clusters and **(E)** cell types. **(F)** Dotplot shows the expression of selected markers of undifferentiated and differentiated spermatogonia for each germ cell subset seen in UMAP plot labelled as SSC, progenitor and differentiating. The size of each dot represents the percentage of cells within a cell type, while the color encodes the average expression level across all cells within a cell type (blue is high).

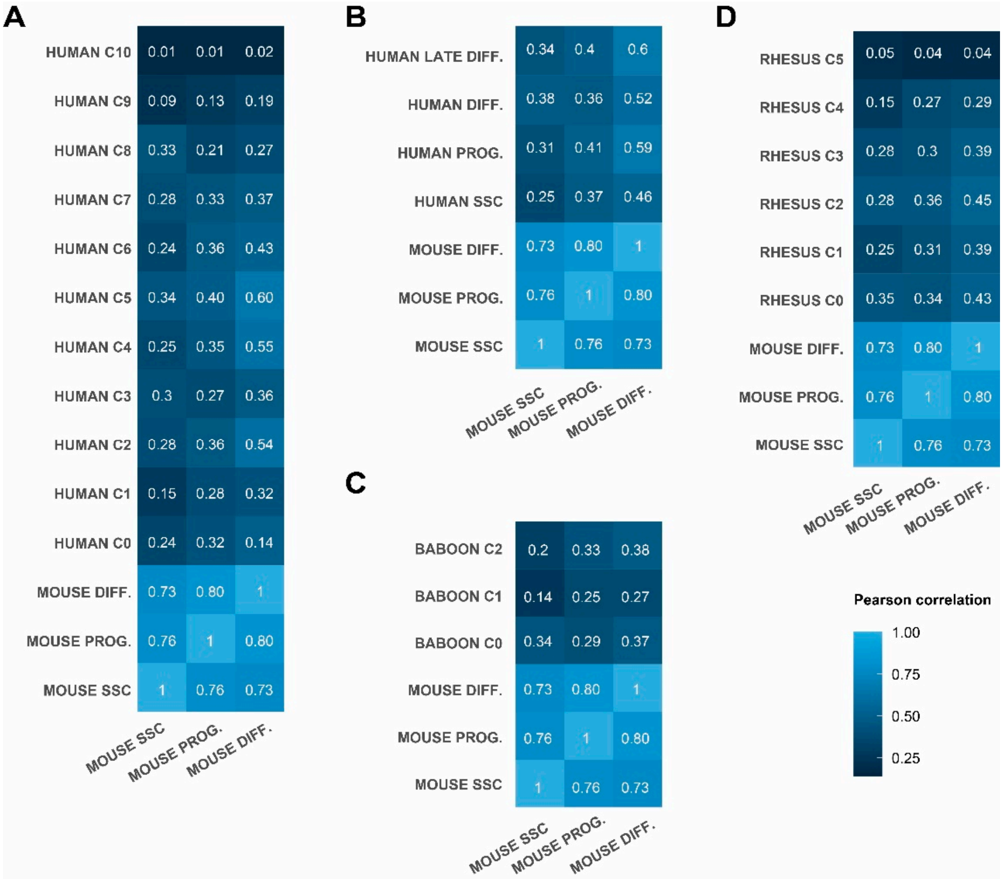

Figure S3. Cross-species comparison of mouse SSCs with human, baboon, and rhesus spermatogonial clusters. (**A**) Correlation heatmap shows the comparison of the mouse SSC cluster with human unbiased spermatogonial clusters (Human C0–10), colored based on the Pearson correlation coefficient. (**B**) Correlation heatmap shows the comparison of the mouse SSC cluster with human spermatogonial cell types (SSC, Progenitor, Differentiating, and Late differentiating). (**C**) Correlation heatmap shows the comparison of the mouse SSC cluster with unbiased baboon germ clusters (Baboon C0–2). (**D**) Correlation heatmap shows the comparison of the mouse SSC cluster with unbiased rhesus germ clusters (Rhesus C0–5).

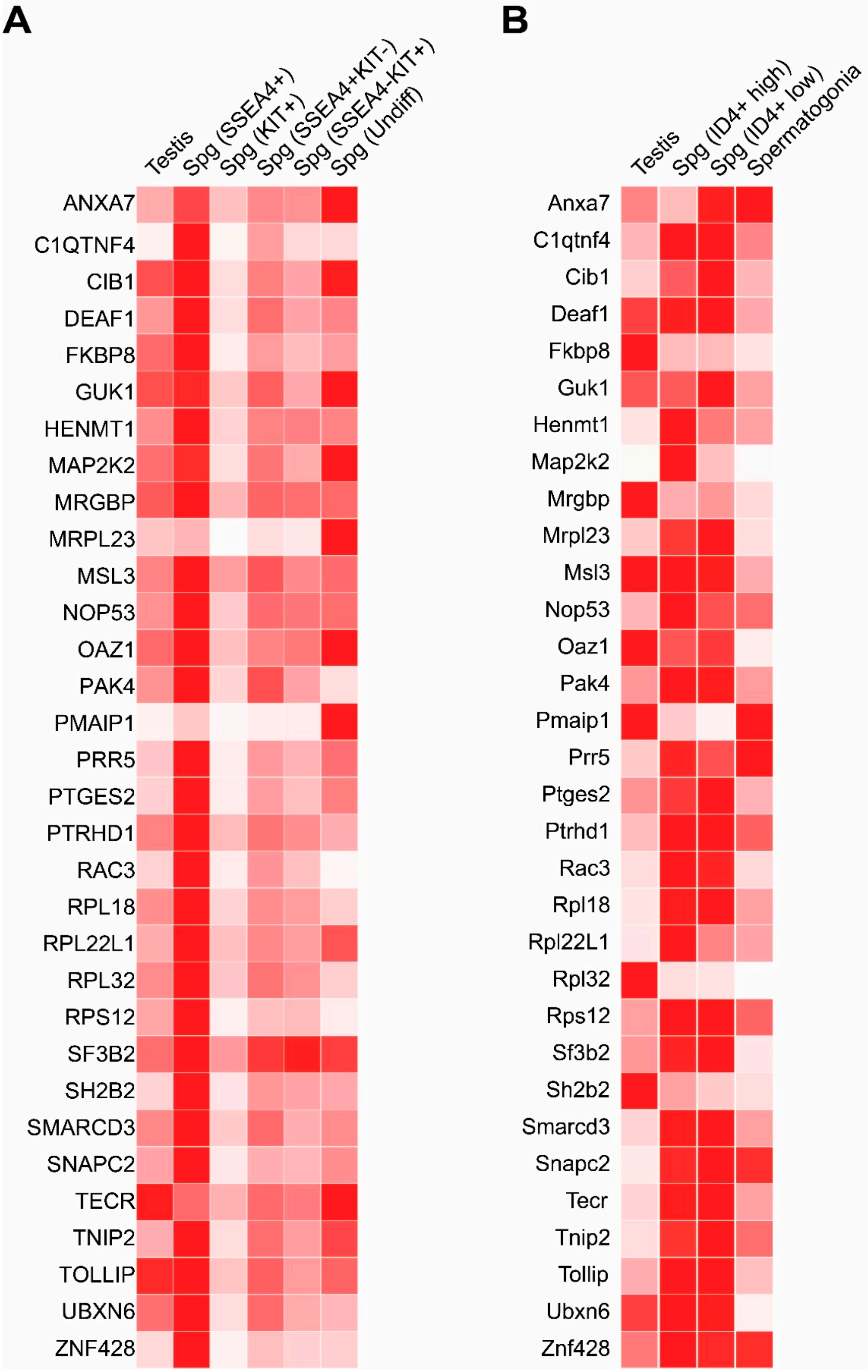

Figure S4. Primate SSC marker expression in published human and mouse RNA-seq datasets. Heatmap shows mRNA expression of 32 genes with elevated expression in the primate SSC clusters from our data in (**A**) human and (**B**) mouse spermatogonia from the Mammalian Reproductive Genetics Database (https://orit.research.bcm.edu/MRGDv2). Red denotes high abundance; white is low abundance. The columns are datasets and the rows are genes.
